# Uncertainty Quantification in the In Vivo Image-Based Estimation of Local Elastic Properties of Vascular Walls

**DOI:** 10.3390/jcdd10030109

**Published:** 2023-03-04

**Authors:** Benigno Marco Fanni, Maria Nicole Antonuccio, Alessandra Pizzuto, Sergio Berti, Giuseppe Santoro, Simona Celi

**Affiliations:** 1 BioCardioLab, Bioengineering Unit, Fondazione Toscana “G. Monasterio”, 54100 Massa, Italy; 2 Pediatric Cardiology Unit, Fondazione Toscana “G. Monasterio”, 54100 Massa, Italy; 3 Adult Cardiology Unit, Fondazione Toscana “G. Monasterio”, 54100 Massa, Italy

**Keywords:** uncertainty quantification, numerical modeling, imaging, fluid–structure interaction, mechanical properties, magnetic resonance imaging

## Abstract

Introduction: Patient-specific computational models are a powerful tool for planning cardiovascular interventions. However, the in vivo patient-specific mechanical properties of vessels represent a major source of uncertainty. In this study, we investigated the effect of uncertainty in the elastic module (*E*) on a Fluid–Structure Interaction (FSI) model of a patient-specific aorta. Methods: The image-based χ-method was used to compute the initial *E* value of the vascular wall. The uncertainty quantification was carried out using the generalized Polynomial Chaos (gPC) expansion technique. The stochastic analysis was based on four deterministic simulations considering four quadrature points. A deviation of about ±20% on the estimation of the *E* value was assumed. Results: The influence of the uncertain *E* parameter was evaluated along the cardiac cycle on area and flow variations extracted from five cross-sections of the aortic FSI model. Results of stochastic analysis showed the impact of *E* in the ascending aorta while an insignificant effect was observed in the descending tract. Conclusions: This study demonstrated the importance of the image-based methodology for inferring *E*, highlighting the feasibility of retrieving useful additional data and enhancing the reliability of in silico models in clinical practice.

## 1. Introduction

In the last decade, the interest in patient-specific numerical modeling has kept spreading in the context of the decision-making process of cardiovascular interventions [1,2,3]. In this view, it has been demonstrated that in silico models can be used to simulate the preoperative scenario at a patient-specific level, representing a valuable tool to stratify risk [4,5], increase diagnostic power [6,7], and inform the planning of interventions [8]. Furthermore, the Food and Drug Administration is strongly supporting the usage of computational simulations in the context of clinical in silico trials for the development of novel devices and treatment strategies, according to the V&V40 statement [9]. However, the effective translation of numerical models into clinical practice still requires several gaps to be covered. The adaptation of cardiovascular models toward reliable in vivo patient-specific conditions represents a major challenge [10], especially due to the multiple sources of uncertainty propagating through the model and affecting the reliability of numerical results [11,12,13,14]. Currently, the implementation of trustworthy in vivo patient-specific mechanical properties in numerical models represents the biggest source of uncertainty [15,16]. In fact, the lack of patient-specific mechanical properties strongly limits the reliability of numerical simulations of cardiovascular interventions. These simulations depend not only on an accurate representation of the patient’s anatomy and related boundary conditions, but also on the mechanical interaction between the device and the implantation site. State-of-the-art imaging techniques permit a precise modelling of the patient’s anatomy as well as reliable functional information such as blood velocity and flow rate [17,18]. Hence, on one hand, image-based data are the main source of input information for the representation of the in vivo conditions. However, on the other hand, the mechanical behavior of in vivo vessels is the main source of uncertainty. The response of the vascular wall to blood flow [19,20,21] or device interaction [8,22] is not only contingent on the intrinsic material properties of the vessel, but it is also related to the properties of the surrounding structures and tissues [23]. In this context, several studies have focused on the extraction of in vivo mechanical properties, mainly coupling inverse computational techniques and imaging [24,25,26]. However, iterative methods are computationally expensive and time-consuming, making them unsuitable to provide rapid clinical feedback. Moreover, these methodologies still require pressure measurements [27,28], which are not always available by clinical routine, besides being invasive. In spite of this, the crucial importance of including in vivo imaging information as a starting point to extract reliable wall characteristics has been highlighted. The availability of enhanced mechanical information, which includes in vivo patient-specific image data, would significantly increment the value of numerical models, thus easing their translation in clinical practice.

Recently, we implemented a novel methodology called the χ-method [29], which provides an enhanced formulation to estimate the elastic module (*E*) of a vessel wall solely from imaging data, without requiring invasive pressure information. The proposed approach was based on the analysis of dynamic information of area deformation and flow variations, which are both easily retrievable from standard functional imaging, such as phase contrast magnetic resonance imaging, routinely acquired in the pre-procedural assessment of the patient before cardiovascular interventions. However, notwithstanding the promising results achieved, with the χ-method significantly increasing the reliability of the output *E* value with respect to the original methodology, a residual error still persisted. In the worst case, a percentage difference of about 20% between the ground truth and the estimation of *E* was observed. In this scenario, computational simulations are a powerful tool to determine the level of robustness of a model with respect to the variation of input parameters [30], such as the *E* value of a vessel wall.

The aim of the present study was to quantify the effects of the residual deviation of the χ-method to estimate *E* in the computational modelling of the hemodynamics and lumen deformations of a patient-specific aorta. The uncertainty quantification in the in vivo estimation of *E* was performed by using the generalized polynomial chaos expansion method [31], which allows us to obtain a continuous response surface in the parameter space, using few deterministic simulations [30,32]. The stochastic results are reported as stochastic standard deviations and probability distribution functions.

## 2. Materials and Methods

The influence of the residual error of the recent χ-method was investigated on a Fluid–Structure Interaction (FSI) model of a patient-specific aorta with a mild aneurysm in the ascending tract. Mechanical properties of the aortic wall, in terms of the elastic module (*E*), were assessed from image data of the patient using the χ-method. A few deterministic FSI simulations were run with different *E* values for the vessel wall by imposing a variation on the uncertain *E* according to previous results. The uncertainty quantification was evaluated on the output variables of flow rate and cross-sectional area deformation along the cardiac cycle. The stochastic surface responses were measured at specific cross-sections of the model.

### 2.1. Imaging Analysis

Numerical modeling of the patient’s aorta was based on the analysis of Computed Tomography (CT, Aquilion ONE, Toshiba, Tokyo, Japan) and Phase Contrast Magnetic Resonance Imaging (PCMRI, Ingenia, Philips Healthcare, Amsterdam, The Netherlands). Patient informed consent was obtained for this study.

**Figure 1:**
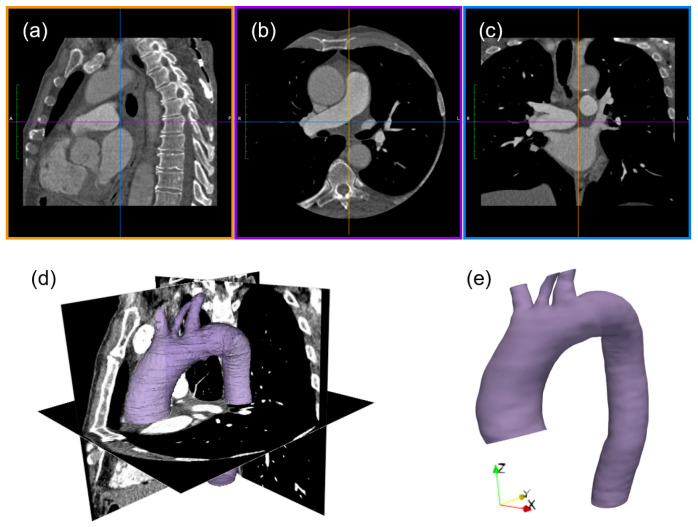
CT imaging processing: sagittal (**a**), axial (**b**), and coronal (**c**) planes of CT dataset, raw segmentation result (**d**), and refined model of the aorta (**e**).

The geometry of the aorta was extracted from the contrast-enhanced CT angiography of the patient (pixel spacing 0.5 mm, slice thickness 1.0 mm). The software 3D Slicer (slicer.org) [33] was used for the CT dataset post-processing. A semi-automatic segmentation algorithm was adopted to extract the vessel anatomy, combining threshold techniques and manual adjustment. Following the segmentation process, the model was refined using the Meshmixer package (Autodesk, Inc., Mill Valley, CA, USA), for the smoothing of the extracted surface. The virtual model consisted of the ascending aorta right after the sinus of Valsalva, the supra-aortic branches, and the descending aorta, as depicted in Figure 1.

Boundary conditions and mechanical properties of the numerical model were evaluated from the patient’s standard PCMRI data acquired at the ascending aorta. Conventional bi-dimensional PCMRI is an established technique for the non-invasive quantitative assessment of blood velocities, segmented and averaged over several consecutive heartbeats at specific sections of the analyzed vessel [34,35,36].

**Figure 2:**
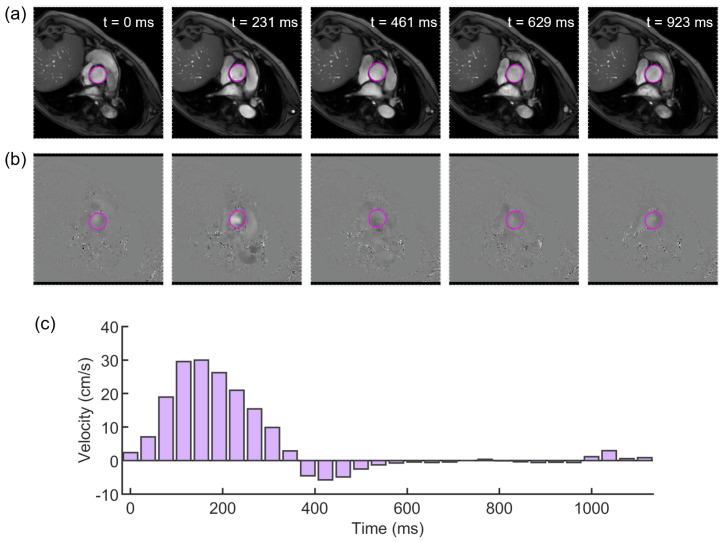
Evenly spaced subsample of PCMRI frames, with aortic lumen countered with ROI (magenta) in both magnitude (**a**) and phase images (**b**); (**c**) flow velocity (magenta boxes) obtained from PCMRI segmentation of the lumen of the aorta.

In this case, the PCMRI dataset consisted of a 30-frame sequence of a cross-section acquired at the level of ascending aorta, proximal to the aortic valve. Segment software (Medviso AB, Sweden) [37] was used to elaborate the PCMRI data. Each frame of the sequence included phase and magnitude images (Figure 2). For each magnitude image, the lumen of the aorta was outlined by adopting a vessel contour-tracking algorithm to select the Region of Interest (ROI), from which the cross-sectional area deformation along the cardiac cycle was measured (Figure 2a). The same ROI was transferred to the phase images (Figure 2b) to extract the patient’s through-plane blood flow velocity (Figure 2c). A second PCMRI dataset, acquired at the descending aorta, was used to validate the numerical simulations by comparing the in vivo and the simulated flow.

### 2.2. Evaluation of the Elastic Module from PCMRI

The *E* value of the in vivo patient-specific aortic wall was inferred by using the recent χ-method, based on the analysis of PCMRI data to calculate the Pulse Wave Velocity (PWV), an established indicator of arterial stiffness [7,38,39].

The PWV of the patient’s aorta was computed from the flow-area (QA) curve as obtainable from the PCMRI segmentation, evaluating flow and area variations along the cardiac cycle from the elaboration of phase and magnitude images, respectively. In fact, the relationship between the cross-sectional area *A* of the vessel and the flow *Q* passing through the same section during the reflection-free early systole period of the cardiac cycle can be approximated as a first-order linear equation [40]. Hence, the PWV is computed as:

(1) $$PWV={\left.\frac{dQ}{dA}\right|}_{\mathrm{early}\;\mathrm{systole}\;}$$

where dA is the incremental variation of the cross-sectional area, and dQ is the incremental variation of the flow passing through the section.

According to our previous studies [29,41], the local *E* value of the vessel wall was demonstrated to be reliably inferred using the following formulation:

(2) $$E=3\;\chi \;\rho \;PWV^{2}\left(1+\frac{A_{0}}{WCSA}\right)$$

in which ρ is the blood density, $A_{0}$ is the vascular lumen area, and WCSA is the wall cross-sectional area (i.e., the area between inner and outer diameter), both measured at diastole. The factor χ, which was not included in the initial formulation [42], was found to be crucial to enhance the validity of the *E* value estimation. It was defined as

(3) χ=γRAC

where RAC is the relative area change along the cardiac cycle, and γ is a function of the cross-sectional diastolic area of the vessel, the flow passing though the analyzed section, and the internal pressure, as described in [41].

However, although the estimation of *E* from Equation (2) was significantly more reliable than the original formulation, in which the factor χ was not included, a residual gap still persisted.

### 2.3. Uncertainty Quantification

In this study, we used the generic Polynomial Chaos (gPC) expansion method to assess the effect of the uncertainties related to the image-based estimation of the *E* of the vessel wall by means of the χ-method on an FSI model of a patient-specific aorta. The uncertainty quantification was evaluated on two FSI output variables, i.e., the flow variations and the area deformation along the cardiac cycle, as measured at specific cross-sections along the aorta’s centerline. The PCMRI-based estimation of the *E* value of the patient’s vessel, indicated as $\hat{E}$, was used as starting value to define the quadrature points. A deterministic FSI simulation was run for each quadrature point.

The gPC is a stochastic expansion method that represents the uncertainty in a system as a linear combination of orthogonal polynomials [31]. The generation of the stochastic response surface is evaluated in three main steps: (i) calculation of the quadrature points $P_{q}$ starting from $\hat{E}$; (ii) running of as many deterministic simulations as quadrature points; (iii) assessment of the gPC for the quantity of interest *X*.

The quantity of interest *X* can be expressed as

(4) $$X\left(\omega \right)=\sum_{r=0}^{\infty }a_{r}\Phi_{r}\left(\zeta \left(\omega \right)\right)$$

where ω is an elementary event, ζ(ω) is the random variable representing the uncertain parameter *E*, $\Phi_{r}$ is the *r*-th orthogonal polynomial basis, and $a_{r}$ is the related coefficient. Given the orthogonality property of the polynomial basis, the coefficient $a_{r}$ is calculated as

(5) $$a_{r}=\frac{\langle X,\Phi_{r}\rangle }{\langle \Phi_{r},\Phi_{r}\rangle }=\frac{1}{\langle \Phi_{r},\Phi_{r}\rangle }\int_{\mathrm{supp}(\zeta )}\Phi_{k}\left(\zeta \right)\eta \left(\zeta \right)\delta \left(\zeta \right)$$

where supp(ζ) is the integration domain, i.e., the support of the random variable ζ, and η(ζ) is the weight function ensuring the orthogonality for the specific polynomial function.

The Probability Distribution Function (PDF) of the studied uncertain parameter (*E* from χ-method) was assumed to be uniform due to the unavailability of statistical information, and the uniform PDF is the least informative distribution able to provide the highest variance in given intervals. Consequently, Legendre polynomials were used as the polynomial basis. Each dimension of the polynomial basis was truncated to the order n=3, thus requiring the conduction of n+1 deterministic simulations. The convergence of the truncated gPC expansion was assessed by checking that the coefficients related to higher order polynomials strongly decreased with respect to the zero order coefficient. The four quadrature points for the simulations were computed based on the deviation from the ground of truth observed in the previous study [29], which was about ±20%. With the aim of considering a safe range of variation of $\hat{E}$, the uncertain parameter was allowed to vary in the range $\hat{E}\in \left[0.77\hat{E},1.23\hat{E}\right]$. In Table 1, we report the computation of the quadrature points following the Gauss–Legendre integration rule [30,32].

### 2.4. In Silico Modeling

The four FSI simulations, one for each quadrature point, of the patient-specific aorta were carried out using the commercial package LS-DYNA R12 (Ansys, Inc., Canonsburg, PA, USA), solving both Computational Fluid Dynamics (CFD) and Computational Solid Mechanics (CSM) for the fluid and structural parts, respectively.

A two-way strong coupling scheme was adopted to simulate the fluid–structure interaction, considering the influence of the blood pressure on the deformable aortic wall and, reciprocally, the effect of wall motion on the fluid domain. An arbitrary Lagrangian Eulerian formulation was used for the mesh deformation. Fluid and structural domains had matching interfaces for an optimal solution exchange.

**Figure 3:**
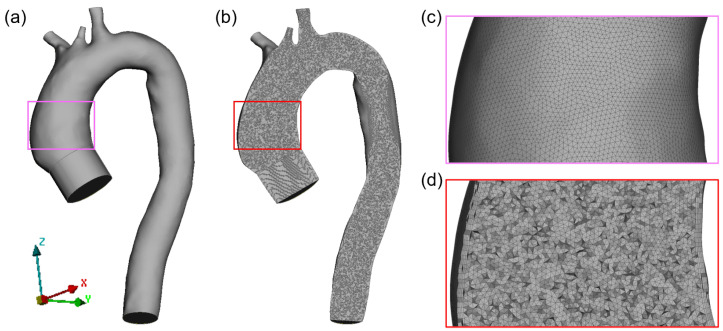
Fluid domain mesh (**a**) and plane cut showing internal elements (**b**) with grid details at wall surface (**c**) and at plane cut (**d**).

A grid mesh of 3,847,788 tetrahedral elements (average size 1.0 mm) with five inflation layers (growth factor 1.2) was generated for the fluid domain (Figure 3) using the ANSA pre-processor software (BETA CAE Systems, Switzerland). An extension was added at the aortic inlet to allow the flow to fully develop. The structural mesh of the vessel wall was generated with two layers among the thickness. A linear elastic isotropic model was used to describe the aortic wall, with a Poisson ratio of 0.49. In order to avoid locking phenomena for nearly incompressible materials, a selective reduced integrated formulation was used as the element formulation of the structural domain [43,44,45]. A preliminary mesh sensitivity analysis was performed to confirm a grid-independent solution. The element size of 1 mm was selected as the best compromise between solution accuracy and computational time (see Appendix A). Four FSI simulations were run with four different *E* values corresponding to the quadrature points (Table 1). Density of the aortic wall was set equal to 1200 kg m${}^{-3}$. The solid domain was bounded with fixed edges at the three supra-aortic branches and descending aorta.

The blood was implemented as an incompressible and Newtonian fluid, with a density of 1060 kg m${}^{-3}$ and a dynamic viscosity of 0.05 kg m${}^{-1}$ s${}^{-1}$. Patient-specific boundary conditions were implemented to simulate a hemodynamic realistic scenario. The flow rate waveform as measured from the patient’s PCMRI was used as the inflow boundary condition at the aortic valve section, while outflow conditions for the descending aorta and the supra-aortic branches were based on the Windkessel model [46], with RCR parameters (namely proximal resistance $R_{p}$, capacitance *C*, and distal resistance $R_{d}$) opportunely tuned to guarantee a physiological pressure waveform at the outlets [6]. The values of the RCR parameters for each outlet are listed in Table 2.

The simulations were performed for 10 cardiac cycles (each lasting 1.12 s according to the patient’s data) to obtain numerical stability and reach a regime state for the Windkessel models. A time-step size of 0.005 s was used for both CFD and CSM solvers.

### 2.5. Post-Processing

Results from the last simulated cardiac cycle were considered for post-processing. Flow and area temporal variations were extracted from five cross-sections CS, perpendicular to the model centerline, along the course of the patient-specific aorta. The open-source software VMTK (Vascular Modeling Toolkit, Orobix srl, Italy) [47] and ParaView (Kitware, Inc., Clifton Park, NY, USA) [48] were used to compute the centerline and to process the FSI results, respectively.

**Figure 4:**
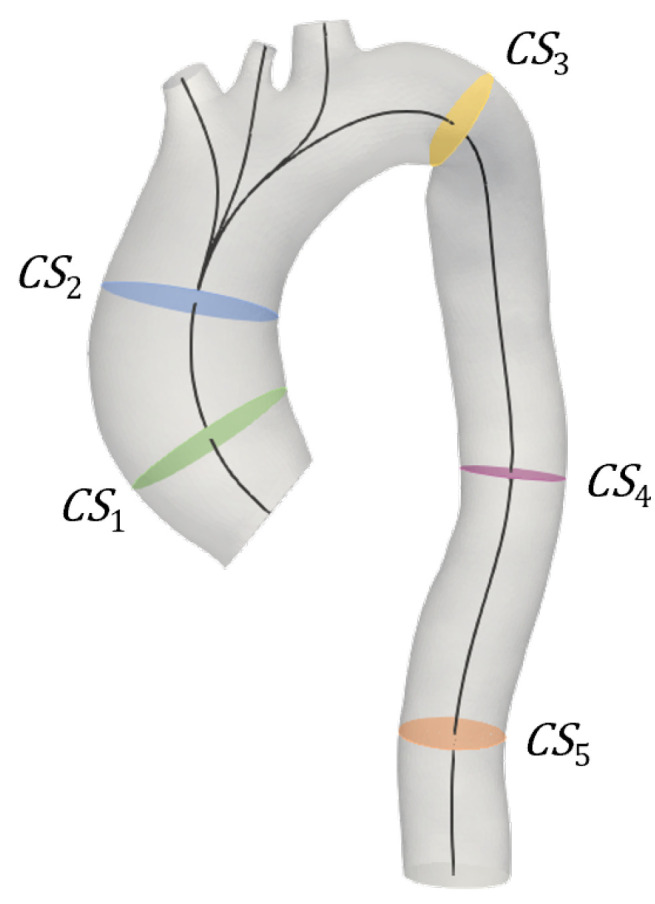
Location of the cross-sections extracted from the FSI simulations, perpendicular to the model centerline: $CS_{1}$ (green) proximal to inlet valve; $CS_{2}$ (blue) at the ascending aorta; $CS_{3}$ (yellow) after supra-aortic branches; and $CS_{4}$ (pink) and $CS_{5}$ (orange) at the descending aorta, with this latter closer to the outlet section.

Figure 4 depicts the location of the five analyzed cross-sections: $CS_{1}$ was extracted proximally to the aortic valve; $CS_{2}$ and $CS_{3}$ were extracted at the aortic arch, before and after the supra-aortic trunks, respectively; and $CS_{4}$ and $CS_{5}$ were extracted at the descending aorta at two different positions, with $CS_{5}$ closer to the outlet.

Separate gPC analyses were conducted using as input the time-dependent flow $Q_{i}$ and area $A_{i}$ curves as extracted from each cross-section $CS_{i}$. The open-source software Dakota (National Technology and Engineering Solutions of Sandia, LLC, United States) [49] was adopted to compute the *i*-th response surface for $Q_{i}$ and $A_{i}$, measuring the uncertainty related to the image-based estimation of $\hat{E}$ and its effects on the output variables from $CS_{i}$.

## 3. Results

**Figure 5:**
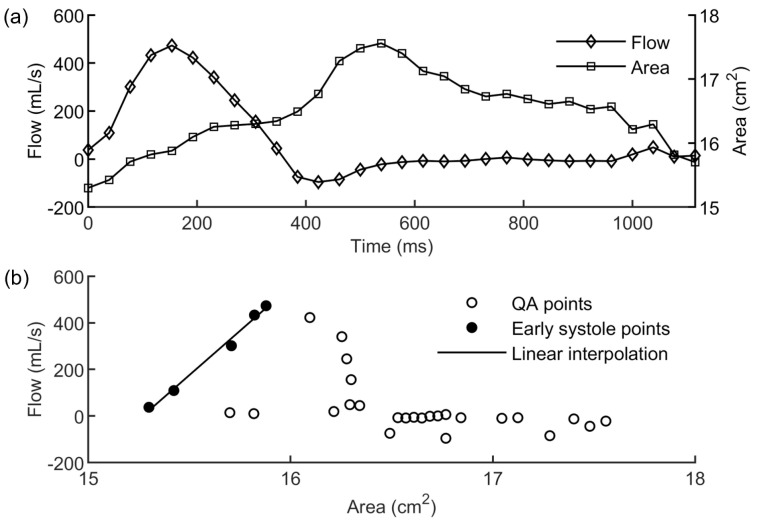
(**a**) Flow and area variations along the cardiac cycle obtained from the segmentation of the patient’s PCMRI data; (**b**) QA loop (white circles) with indication of the points belonging to the early systole period (black circles) used for the linear interpolation (black line) to compute the PWV.

The first result was represented by the $\hat{E}$ value computed from the patient’s PCMRI data using the χ-method. Figure 5a depicts the flow and area variations along the cardiac cycle obtained from the PCMRI segmentation. The PWV was estimated from the resulting QA loop (Figure 5b) using Equation (1).

Finally, the patient-specific $\hat{E}$ of the aortic wall was computed using Equation (2), resulting in 2.02 MPa. Hence, the four quadrature points were equal to 2.42 ($x_{1}$), 2.18 ($x_{2}$), 1.86 ($x_{3}$), and 1.62 ($x_{4}$) MPa, according to Table 1, with each value assigned as *E* of the aortic wall in the deterministic FSI simulations.

**Figure 6:**
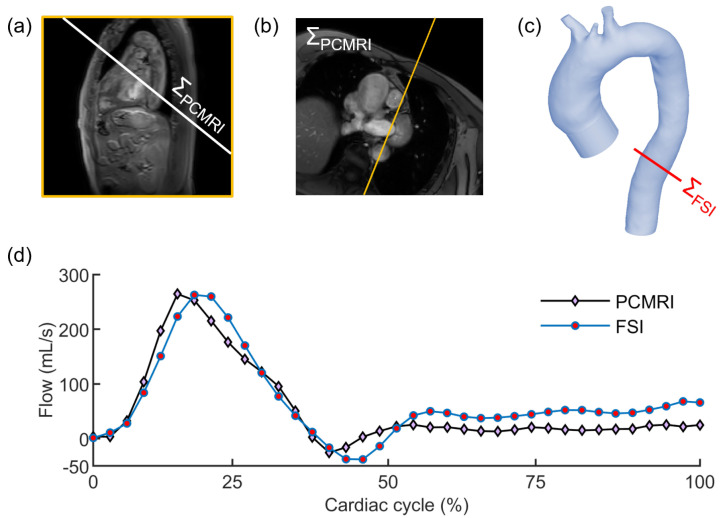
Localizer image (**a**) used to define the acquisition plane $\Sigma_{PCMRI}$ of the PCMRI at the descending aorta (**b**) and equivalent plane $\Sigma_{FSI}$ in the FSI domain (**c**); (**d**) comparison between flow curves as extracted from PCMRI data of the patient (black line with diamond markers) and FSI simulation (blue line with circle markers) at the same plane of the descending aorta.

To quantify the reliability of the FSI simulations, the flow extracted from the PCMRI dataset of the patient at the descending aorta was compared to the simulated flow at the same plane of the imaging, as depicted in Figure 6. Along the entire cardiac cycle, the error between the simulated and the patient’s flow curves was 1.39 ± 2.27% (N = 37). Considering only the systolic period, the error was found to decrease up to 0.52 ± 0.77% (N = 7). Such low deviation from the in vivo data demonstrated the correct implementation of the patient-specific aortic scenario, including settings of RCR parameters that are crucial to simulate reliable hemodynamic conditions. The comparison was given for a representative simulation. As proved in the following, the flow results among the four FSI simulations were highly comparable, especially in the descending aorta tract.

**Figure 7:**
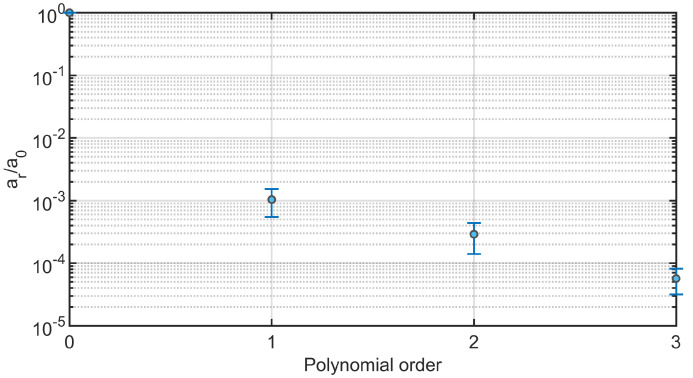
Mean and standard deviation computed along the selected cross-sections of the coefficient ratio $a_{r}/a_{0}$ of the gPC polynomial expansion for the performed stochastic analyses.

**Figure 8:**
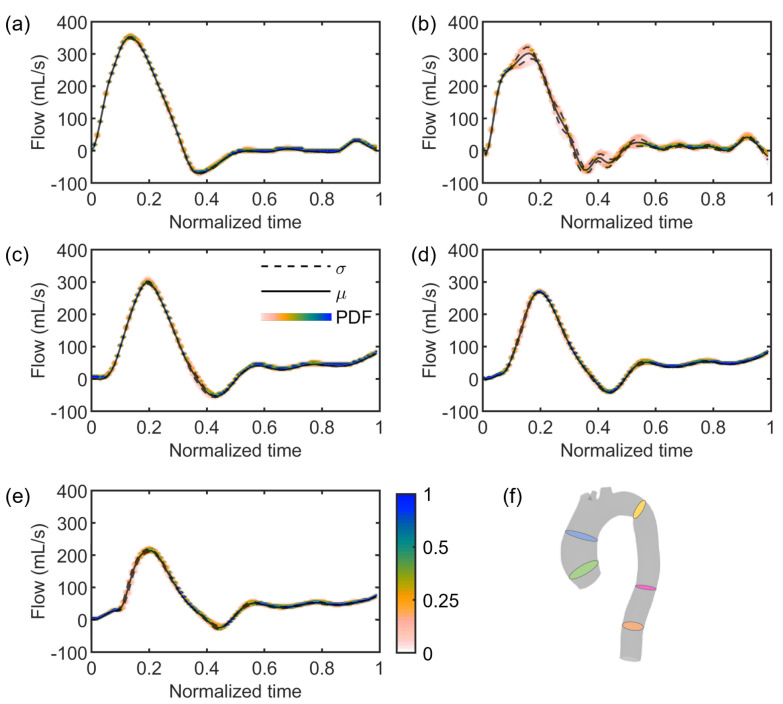
PDF (color scale), mean (straight line), and standard deviation (dashed line) plots of flow variations along the cardiac cycle at cross-sections $CS_{1}$ (**a**), $CS_{2}$ (**b**), $CS_{3}$ (**c**), $CS_{4}$ (**d**), and $CS_{5}$ (**e**), respectively identified by the green, blue, yellow, pink, and orange slices in the aortic model (**f**).

**Figure 9:**
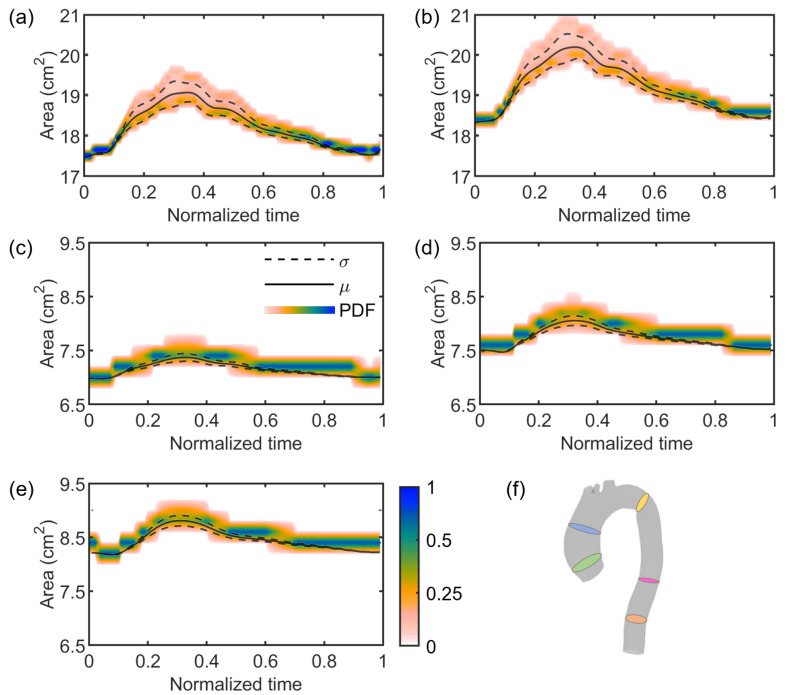
PDF (color scale), mean (straight line), and standard deviation (dashed line) plots of area variations along the cardiac cycle at cross-sections $CS_{1}$ (**a**), $CS_{2}$ (**b**), $CS_{3}$ (**c**), $CS_{4}$ (**d**), and $CS_{5}$ (**e**), respectively identified by the green, blue, yellow, pink, and orange slices in the aortic model (**f**).

Regarding the stochastic analysis, four deterministic simulations were run, based on the assumption that a truncation of the gPC expansion to the third order was sufficient, as demonstrated in Figure 7. Following this verification, the results from gPC analyses were extracted in terms of PDF, mean μ, and standard deviation σ plots related to each flow $Q_{i}$ and area $A_{i}$ time-dependent curve extracted from each cross-section $CS_{i}$. In Figure 8 and Figure 9, we reported the results related to flow and area variations, respectively. Regarding the PDFs, blue values in the color scale represent the most probable value to occur for each time instance. On the other hand, the less probable values are indicated in white. The flow rate was found to present an insignificant dependence on the uncertain $\hat{E}$ parameter (Figure 8). For all CS, the analyzed statistical quantities were found to minimally vary along the cardiac cycle. A slight exception was noticed for $CS_{2}$, in which wider distributions at end-systolic and early-diastolic periods were observed (Figure 8b). Following the gPC analysis, the area variation (Figure 9) was more affected by the $\hat{E}$ uncertainty than flow rate. In particular, the widest PDF and statistical distributions of area variation were observed at $CS_{1}$ (Figure 9a) and $CS_{2}$ (Figure 9b), in accordance with the higher values of cross-sectional area in the ascending aorta. High data uncertainty was noted in the zones of area peak and maximum incremental variation, i.e., during the periods of increasing and decreasing of area deformation in the heart beat. While such zones presented high variation, the uncertainty strongly decreased in the time intervals corresponding to the early systole and late diastole, manifesting a low variation.

**Figure 10:**
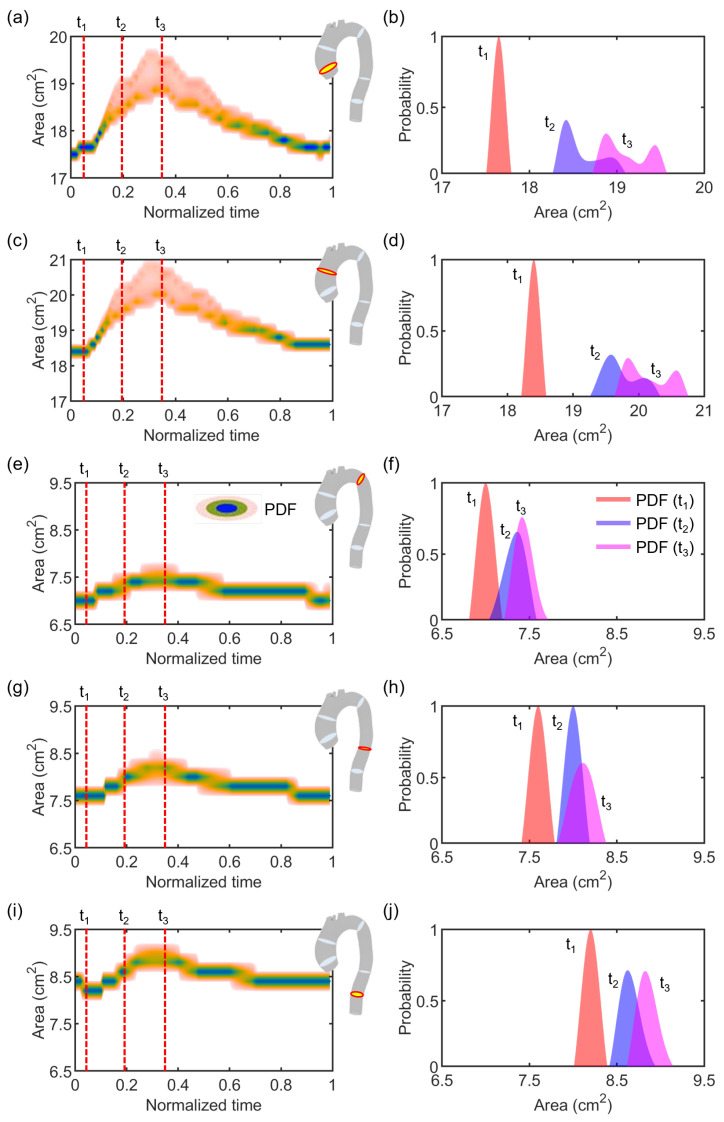
PDFs of area variation at the five cross-sections, i.e., $CS_{1}$ (**a**,**b**), $CS_{2}$ (**c**,**d**), $CS_{3}$ (**e**,**f**), $CS_{4}$ (**g**,**h**), and $CS_{5}$ (**i**,**j**) along the entire cardiac cycle (**a**,**c**,**e**,**g**,**i**) and at the time instances $t_{1}$, $t_{2}$, and $t_{3}$ of the cardiac cycle (**b**,**d**,**f**,**h**,**j**).

Figure 10 reports a few examples of probability distributions for the five cross-sections at three different time instances of the cardiac cycle, i.e., when systole starts ($t_{1}$), at early systole ($t_{2}$), and at systolic peak ($t_{3}$). Similarly to the time-dependent PDFs, the distributions at the selected fixed times of cross-sections extracted from the ascending aorta, i.e., $CS_{1}$ and $CS_{2}$, were highly comparable. In particular, PDF($t_{1}$) presented a single centered lobe for both $CS_{1}$ and $CS_{2}$, while PDF($t_{2}$) and PDF($t_{3}$) were characterized by a double-lobed shape (Figure 10b–d), likely due to the significantly higher variability observed at these times, which may arise from the secondary flows developing at the ascending aorta during late systole [50,51,52]. On the contrary, narrow distributions were instead observed at $CS_{3}$, $CS_{4}$, and $CS_{5}$ of the descending aorta (Figure 9c–e), which is known to be characterized by a more regular flow. This is confirmed also when looking at the distributions at the fixed times, characterized for these three sections by a single mode (Figure 10f,h,j). Hence, a global low variation was observed at $CS_{3}$, $CS_{4}$, and $CS_{4}$, confirming them to be barely affected by the uncertainty introduced by the image-based estimation of $\hat{E}$ of the vascular wall.

## 4. Discussion

The methodologies presented in this work were entirely based on image data, from the generation of virtual geometry from CT to the definition of boundary conditions and mechanical properties from PCMRI. The χ-method [29] was adopted to compute the patient-specific $\hat{E}$ value of the aortic wall from imaging. This technique relies on the computation of the PWV, which is considered a relevant clinical marker of vessel status [53,54]. A unique requirement of this method is functional imaging of the patient, such as PCMRI or Doppler echocardiography, without using any invasive pressure data, unlike other available methodologies [27,28,55]. The χ-method relies on the measurements of flow variation and an accurate assessment of vessel motility. This latter assessment is crucial input information for the χ-method, which limits the estimation of vascular wall stiffness at specific vessel cross-sections. Usually, just a single cross-section is acquired in clinical practice, depending on the clinical scenario of the patient. Hence, an approximation is made when we extend the local stiffness measured at a given cross-section to the entire vessel. This is a limitation of the presented method given by the actual available clinical imaging data. The 4D Flow MRI technique may represent an alternative, but its actual low spatial resolution strongly limits the reliability of the evaluation of area deformation. Moreover, 4D Flow MRI is not yet part of a standard clinical exam.

The χ-method therefore represents a non-invasive and direct image-based tool to infer local patient-specific elastic properties of in vivo vessel walls. The knowledge of the vessel elasticity at a patient-specific level can provide many clinical advantages. It can provide insight into its structural integrity, is strongly related to the patient’s age [56,57], and can also be used as a bio-marker for disease progression [58,59]. Moreover, being an in vivo measure, the estimated *E* is comprehensive of the patient-specific context of the analyzed vessel, such as the surrounding tissue and anatomical boundaries, thus making the χ-method suitable for the pre-planning phase of cardiovascular interventions using numerical simulations. The possibility of directly measuring the *E* value of a vessel in its in vivo environment from imaging represents a step forward for patient-specific numerical modeling as a clinical instrument. The results from simulations of cardiovascular interventions would benefit from the implementation of a more reliable mechanical interaction between the implanted device and the patient-specific implantation site, which would correspond to the overall in vivo scenario of the patient’s vessel of interest.

In view of this, in this study, we performed an uncertainty quantification of the effect of the intrinsic error still present in the methodology in the estimation of the *E* value of the vessel wall. The gPC technique was adopted to quantify the time-dependent probability distributions of flow and area variations at different cross-sections of the patient-specific aortic model. The adoption of a stochastic analysis based on the gPC expansion theory permitted us to contain the computational cost by requiring few deterministic simulations to run, unlike previous studies performing a larger number of simulations [60]. In the cardiovascular field, the gPC method was already used in previous studies to quantify the effect of uncertain input parameters on model outputs, from boundary conditions in CFD simulations [30,32] to electrophysiology [61] and cardiac pulse propagation [62]. The mutual effect of inflow conditions and arterial stiffness on pulse wave propagation was evaluated by Brault et al. [63], pointing out the importance of flow shape and reflection features.

In the present work, the focus was on the single effect of a comprehensive image-based estimation of vascular stiffness, namely $\hat{E}$ for the studied patient-specific aorta, by imposing the maximum variation of the parameter according to previous findings [29]. Stochastic analyses (Figure 8, Figure 9 and Figure 10) demonstrated a restrained but still present influence of the uncertain $\hat{E}$ value on the investigated output variables, i.e., flow rate and area variations through time at selected sections of the model, indicated in Figure 4. In particular, the effect of uncertainty on flow waveforms was negligible among all the cross-sections, as evidenced by the low-variation distributions depicted in Figure 8. By contrast, a relatively greater effect was measured on area variations, although this was limited to cross-sections of the ascending aorta (Figure 9a,b), corresponding to a larger vessel lumen. In fact, the effect of $\hat{E}$ oscillation on cross-sectional areas belonging to the smaller descending tract was significantly reduced (Figure 9c–e).

The residual error of the χ-method definitely has an impact on the mechanical modeling of vascular walls. Although the flow rate was basically unaffected by the variation of $\hat{E}$, the area was found to be more correlated to wall stiffness at larger lumen, as depicted in Figure 9 and highlighted in Figure 10, where different PDFs at relevant time instances are reported.

Future investigation will be conducted to overcome the limitations of the present work and enhance the reliability of the χ-method. First, a single-case study of a patient-specific aorta was presented. Despite the conduction of a stochastic analysis using the gPC expansion that allowed us to explore a continuous range of *E* values in a realistic range of wall stiffnesses of aorta [32,64,65], additional control cases will be studied. The inclusion of more vascular anatomies from more patients will permit the quantification of the impact of the anatomical variability of vessels and their stiffness with respect to sex, age, and patient clinical history. Pulmonary arteries will be also modelled, considering their different wall stiffness with respect to the aortic wall [66,67]. A second limitation is that the presented method provides an estimation of Young’s module, which may not fully capture the complex, hyperelastic, and anisotropic mechanical properties of vascular tissue [20,68,69]. Nonetheless, the *E* value computed by the χ-method not only reflects the local stiffness of the vessel wall, but it is also inclusive of the mechanical response of the surrounding tissue and anatomical constraints. Hence, despite the assumption of linear elasticity, the extracted *E* value provides a novel comprehensive understanding of the specific and overall context of the patient-specific vessel. However, future objectives of the research will include evolution from the isotropic linear elastic material to more complex models, given the numerical approach used to define the parameters of the χ-method formulation. The same workflow will be applied to infer additional mechanical parameters, including the hyperelastic and anisotropic features typical of vessels, uniquely provided by imaging analysis. The stochastic analysis will be extended to further output variables like wall shear stress and vorticity indexes. Moreover, finite element simulations of minimally invasive interventions, such as transcatheter aortic valve implantation or percutaneous pulmonary valve implantation, will be carried out to quantify the effect of uncertainties in a controlled intraoperative scenario. Furthermore, considering a bottom-up approach, in vitro models will be developed to investigate the effect of uncertainties in a less controllable environment, using 3D printed phantoms of vascular anatomies [70,71] and experimental mock circulatory loops [72,73,74].

In conclusion, the development of new methods and techniques for incorporating trustworthy in vivo patient-specific mechanical properties into numerical models represents an important focus for future work. By improving the accuracy and reliability of these models, outcomes of cardiovascular interventions could be predicted in advance, thus easing the translation of numerical simulations into clinical practice. Moreover, the use of reliable digital twins could help in the validation process of new medical devices and guide their design and development, leading to more effective and safer treatments for patients.

## Figures and Tables

**Table 1:** List of the four quadrature points $x_{i}$, calculated using the Gauss–Legendre integration rule.

Quadrature Points $x_{i}$			
$x_{1}$	$x_{2}$	$x_{3}$	$x_{4}$
$1.2\hat{E}$	$1.08\hat{E}$	$0.92\hat{E}$	$0.8\hat{E}$

**Table 2:** RCR values of the Windkessel models for the boundary conditions assigned to the aorta outlets: the three supra-aortic vessels, i.e., the brachiocephalic artery, the left common carotid artery, and the left subclavian artery, and the descending aorta.

	$R_{p}$ (Kg s${}^{-1}$ m${}^{-4}$)	*C* (m${}^{3}$ Pa ${}^{-1}$)	$R_{d}$ (Kg s${}^{-1}$ m${}^{-4}$)
Brachiocephalic artery	$1.3\times 10^{7}$	$1.5\times 10^{-9}$	$1.3\times 10^{9}$
Left common carotid artery	$5.1\times 10^{7}$	$3.8\times 10^{-10}$	$5.0\times 10^{9}$
Left subclavian artery	$1.1\times 10^{7}$	$1.7\times 10^{-9}$	$1.1\times 10^{9}$
Descending aorta	$2.5\times 10^{6}$	$7.7\times 10^{-9}$	$2.4\times 10^{8}$

**Figure A1:**
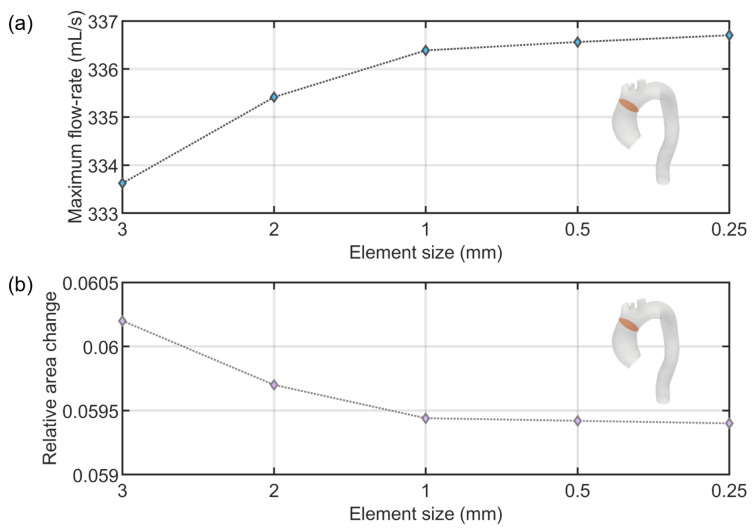
Mesh sensitivity analyses of five meshes (3, 2, 1, 0.5, and 0.25 mm) conducted on relative area change (**a**) and maximum flow rate (**b**) measured at a specific model’s cross-section.

## Data Availability

Not applicable.

## Appendix A. Mesh Sensitivity

Here we report the results of the mesh convergence study conducted on five meshes, considering an element size of 3 mm, 2 mm, 1 mm, 0.5 mm, and 0.25 mm. The sensitivity analyses were conducted on the relative area change and maximum flow rate, which are crucial variables in the methodologies described in our work. Figure A1 depicts the results of the mesh sensitivity analysis. The percentage difference between results from the coarsest (3 mm element size) and finest (0.25 mm element size) meshes measured 0.9% and 1.4% for maximum flow and area variation, respectively, and 0.09% and 0.07% when comparing the used mesh (1 mm element size) and the finest mesh. Hence, the mesh with 1 mm was selected for the study.
